# P190B RhoGAP Overexpression in the Developing Mammary Epithelium Induces TGFβ-dependent Fibroblast Activation

**DOI:** 10.1371/journal.pone.0065105

**Published:** 2013-05-22

**Authors:** Melissa Gillette, Kristi Bray, Alisa Blumenthaler, Tracy Vargo-Gogola

**Affiliations:** 1 Department of Biological Sciences and the Harper Cancer Research Institute, University of Notre Dame, Notre Dame, Indiana, United States of America; 2 Department of Biochemistry and Molecular Biology, Indiana University School of Medicine and the Simon Cancer Center, South Bend, Indiana, United States of America; University of Birmingham, United Kingdom

## Abstract

Rho GTPases mediate stromal-epithelial interactions that are important for mammary epithelial cell (MEC) morphogenesis. Increased extracellular matrix (ECM) deposition and reorganization affect MEC morphogenesis in a Rho GTPase-dependent manner. Although the effects of altered ECM on MEC morphogenesis have been described, how MECs regulate stromal deposition is not well understood. Previously, we showed that p190B RhoGAP overexpression disrupts mammary gland morphogenesis by inducing hyperbranching in association with stromal alterations. We therefore hypothesized that MEC overexpression of p190B regulates paracrine interactions to impact fibroblast activation. Using a combination of in vivo morphometric and immunohistochemical analyses and primary cell culture assays, we found that p190B overexpression in MECs activates fibroblasts leading to increased collagen, fibronectin, and laminin production and elevated expression of the collagen crosslinking enzyme lysyl oxidase. Phosphorylation of the TGF-β effector SMAD2 and expression of the TGF-β target gene *αSma* were increased in p190B-associated fibroblasts, suggesting that elevated TGF-β signaling promoted fibroblast activation. Mechanical tension and TGF-β cooperate to activate fibroblasts. Interestingly, active TGF-β was elevated in conditioned medium from p190B overexpressing MECs compared to control MECs, and p190B overexpressing MECs exhibited increased contractility in a collagen gel contraction assay. These data suggest that paracrine signaling from the p190B overexpressing MECs may activate TGF-β signaling in adjacent fibroblasts. In support of this, transfer of conditioned medium from p190B overexpressing MECs onto wildtype fibroblasts or co-culture of p190B overexpressing MECs with wildtype fibroblasts increased SMAD2 phosphorylation and mRNA expression of ECM genes in the fibroblasts when compared to fibroblasts treated with control CM or co-cultured with control MECs. The increased ECM gene expression and SMAD2 phosphorylation were blocked by treatment with a TGF-β receptor inhibitor. Taken together, these data suggest that p190B overexpression in the mammary epithelium induces fibroblast activation via elevated TGF-β paracrine signaling.

## Introduction

Stromal-epithelial interactions are critical for mammary gland development, and soluble and mechanical cues originating in the stroma impact MEC morphogenesis [1]. Accumulating data have demonstrated that aberrant extracellular matrix (ECM) deposition and reorganization disrupt mammary epithelial morphogenesis and facilitate tumor formation [2], [3]. Stromal fibroblasts become activated during the development and progression of cancer and acquire pro-tumorigenic properties, including elevated secretion of cytokines and growth factors and increased ECM deposition and remodeling [4], [5]. Although the effects of matrix rigidity and cancer-associated fibroblasts on epithelial morphogenesis and tumor formation have been described, the mechanisms by which the epithelium regulates fibroblast activity and ECM production during normal and neoplastic mammary gland development are not well understood.

Rho GTPases have been shown to be critical mediators of interactions between the stromal and epithelial compartments during normal tissue morphogenesis and tumor formation [6]. Rho GTPases, including RhoA, Rac, and Cdc42, are overexpressed and hyperactivated in human breast tumors [7], [8] (for review see [9]), and aberrant Rho GTPase activity has long been known to promote transformation, proliferation, motility, and invasion [10]. More recently, the Rho signaling network has been shown to be a critical mediator of mechanotransduction between the stromal and epithelial compartments during normal tissue morphogenesis and tumor formation [6]. For example, ECM rigidity increases activation of RhoA and its downstream effector Rho kinase (ROCK) to drive aberrant proliferation and disrupt epithelial architecture, both of which contribute to tumor development and progression [11], [12], [13]. Targeted overexpression of ROCK in the epidermis increases actomyosin contractility leading to stromal activation, increased collagen density and ECM stiffness, and tumor formation [14]. These studies suggest that Rho signaling affects tissue morphogenesis and homeostasis by regulating a bidirectional mechanical signaling loop between the stromal and epithelial compartments. Although RhoA and ROCK have been shown to be important regulators of mechanical signaling, the contribution of other Rho family GTPases and their regulators is unclear.

Rho GTPase activity is dependent on GTP loading and hydrolysis rates, which are tightly regulated in a spatio-temporal manner by positive and negative regulators. P190B Rho GTPase activating protein (GAP), an important regulator of Rac and RhoA, functions as a negative regulator by accelerating GTP hydrolysis [15], [16]. Our previous studies have shown that p190B plays a crucial role in the developing embryonic and postnatal mammary gland [17], [18], and intriguingly, that it has pro-tumorigenic functions during MMTV-Neu induced mammary tumor formation [19], [20]. Furthermore, we have demonstrated that p190B overexpression in the developing mammary gland promotes aberrant terminal end bud (TEB) morphogenesis and hyperbranching in association with alterations in the adjacent stroma [18]. Here we aimed to define the mechanisms by which p190B overexpression in the mammary epithelium induces stromal alterations.

## Materials and Methods

### Mice

TetO-p190B/MMTV-rtTA (p190B overexpressing) mice [18], MMTV-rtTA (control) mice [21], and FVB mice (wildtype) were used for these studies. All studies were approved by the Institutional Animal Care and Use Committee at the University of Notre Dame and Indiana University School of Medicine (Protocol Number 14-015) and were conducted in accordance with the guidelines of the U.S. Public Health Service Policy for Humane Care and Use of Laboratory Animals. All efforts were made to minimize suffering of the mice. To induce p190B transgene expression and to control for any effects of doxycycline (Dox), p190B overexpressing and control mice were fed Dox containing chow (2 g/kg) (Bio-Serv, S3893) for 7 days.

### Mammary epithelial cell and fibroblast isolation for cell culture, protein lysates, and qRT-PCR Super Arrays

Five to six week-old p190B overexpressing and control mice were treated with Dox diet for 7 days prior to euthanasia and mammary gland dissection. The two, three, and four mammary gland pairs were dissected, and lymph nodes were removed from the number 4 glands. The glands were manually minced and incubated in DMEM/F-12 (Thermo Scientific, SH30272) with 2 mg/ml Collagenase A (Roche, 10103578001), 100 units/ml hyaluronidase (Sigma, H3506), and 1X Antibiotic-Antimycotic (Invitrogen, 15240-062) for 1 hour at 37°C with 200 rpm rotation at a 45° angle. The tissues were shaken manually at 30 and 60 minutes during the digestion to aid in dissociating the tissues.

For MEC isolation, the cells were centrifuged at 600 g for 1 minute at 4°C, washed with wash buffer containing DMEM/F-12 with 5% FBS (JRScientific, 43602) and 1% Antibiotic-Antimycotic, and spun 3 times at 600 g, once for 1 minute and twice for 2 seconds. The cells were washed with PBS and trypsinized in 0.05% Trypsin-EDTA (Invitrogen, 25200) for 5 minutes at 37°C with 200 rpm rotation at a 45° angle. The trypsin was neutralized with wash buffer and the cells were passed through a 40 µm cell filter. The cells were plated in MEGM BulletKit growth media (Lonza, CC-3151) with 2 µg/ml Dox (Clontech, 8634-1) on 2% Growth Factor Reduced Matrigel (BD Biosciences, 354230) coated plates for 48–72 hours at 37°C and 5% CO_2_ before being used for conditioned medium or co-culture experiments or cryopreserved in FBS containing 10% DMSO. Glands from 3 to 5 mice per genotype were pooled when isolating MECs.

For organoid and fibroblast isolation, the cells were centrifuged at 450 g for 10 minutes at 4°C, washed with DMEM/F-12 and centrifuged at 450 g for 10 minutes. The cells were incubated at room temperature with manual shaking in DMEM/F-12 with 2 units/ml DNaseI (Sigma, D2463) and centrifuged at 450 g for 10 minutes. Differential centrifugation was used to separate fibroblasts from organoids. This consisted of pulse centrifugation to 450 g with the supernatant from the first spin containing the fibroblasts. Fibroblasts and organoids for Western analysis were immediately frozen once isolated. Prior to being used for conditioned medium or co-culture experiments, fibroblasts were plated for 48–72 hrs in basic growth media (DMEM/F-12 with 5 µg/ml insulin, 1 µg/ml hydrocortisone, 5 ng/ml EGF, 1% Antibiotic-Antimycotic, and 5% FBS) on Matrigel coated plates at 37°C and 5% CO_2._ Glands from 3 to 5 mice per genotype were pooled when isolating organoids and fibroblasts for protein lysates and culture.

For fibroblast isolation for qRT-PCR Super Arrays, fibroblasts were co-cultured with MECs for 72 hours on Matrigel coated plates in MEGM BulletKit growth media and 2 µg/ml Dox. The MECs and fibroblasts were then separated by differential trypsinization. After washing the cultures with PBS, the cultures were incubated in 0.05% trypsin-EDTA at 37°C for 3–5 minutes. The fibroblast population became unattached during the incubation while the MECs remained attached. The fibroblasts were collected and used to isolate RNA for qRT-PCR Super Arrays.

### Primary fibroblast culture for qRT-PCR, Western analysis, and immunostaining

Control and p190B-associated fibroblasts were plated at 1.5 million cells per Matrigel coated 10 cm plate overnight before washing the cultures with PBS twice and changing the media to minimal media (DMEM/F-12 with 1% ITS) (Cellgro, 25-800-CR) containing 1x Antibiotic-Antimycotic and SB431542 TGFβ receptor inhibitor (Sigma, 4317) in DMSO or DMSO. After 24 hours, the fibroblasts were harvested for qRT-PCR and protein analysis by Western blot. Fibroblasts were pooled from 4 mice/genotype. For evaluation of fibroblast purity, fibroblasts were suspended in basic growth media and plated at a density of 20,000 cells/well in Matrigel coated 8-well chamber slides. The cultures were incubated for 24 hours at 37°C and 5% CO_2_. The cells were fixed in 4% PFA for 15 minutes, washed 3 times with PBS, permeabilized in 0.5% triton X-100 (Fischer Scientific, BP151-500) for 10 minutes, blocked in 5% BSA (Sigma, A7906), 0.5% tween-20 (Fisher Scientific, BP337-500) in PBS with 10% goat serum (Sigma, G9023) for 1 hour, and incubated overnight in a humid chamber with primary antibody diluted 1∶500 in blocking buffer. The following day, the cells were washed 3 times with PBS, incubated with secondary antibody diluted 1∶1000 in blocking buffer for 45 minutes, washed 3 times with PBS, incubated in TO-PRO3 1∶200 (Invitrogen, T3605) in PBS for 15 minutes, and mounted with Vectashield with DAPI (Vector Laboratories, H-1200). Primary antibodies included: Keratin 14 (K14) (Convance, PRB-155P) and α smooth muscle actin (Sigma, A5228). Secondary antibodies included anti-rabbit Alexa Fluor 555 and anti-mouse Alexa Fluor 488 (Invitrogen, A21428 and A11001). Images were captured at 200×magnification using a Zeiss Axioimager A1 epifluorescence microscope. Six fields were selected using the DAPI channel and imaged per chamber. The percentage of K14 positive cells was quantified, and at least 500 cells per genotype were counted per experiment. The data represent the average of 2 independent experiments.

### Conditioned medium experiments

Control and p190B-associated fibroblasts and control and p190B overexpressing MECs were plated in basic growth media (fibroblasts) or MEGM growth media with 2 µg/ml Dox (MECs) until they reached 80% confluency. The cultures were washed twice with PBS and the media was changed to minimal media containing DMEM-F/12, 1% ITS (Cellgro, 25-800-CR), and 1X Antibiotic-Antimycotic. MEC cultures were also treated with 2 µg/ml Dox. The media was collected after 48 hours and concentrated using Amicon® Ultra 3K Centrifugal Filter Units (Millipore, UFC200324 and UFC900324) for protein analysis or treatment of wildtype fibroblasts. Wildtype fibroblasts were grown to 80% confluency in basic growth media, washed twice with PBS, and treated with concentrated conditioned media diluted back to 1×strength with minimal media for 48 hours at 37°C and 5% CO_2_. After 48 hours, the fibroblasts were either harvested for protein analysis by Western blot or gene expression analysis by qRT-PCR.

### Co-culture experiments

Control and p190B overexpressing MECs were plated in basic growth media with 2 µg/ml Dox for 48 hours prior to addition of wildtype fibroblasts at a concentration of 2.5∶1. The co-cultures were incubated for 72–96 hours at 37°C and 5% CO_2_ in basic growth media with 2 µg/ml Dox and 5 µM SB431542 TGF-β receptor inhibitor in DMSO (Sigma S4317) or DMSO. The fibroblasts were removed by differential trypsinization with a 3-5 minute incubation at 37°C in 0.05% trypsin-EDTA for protein analysis by Western blot or gene expression analysis by qRT-PCR.

### Tissue preparation, immunostaining, and analysis

Five-week old control and p190B overexpressing mice were treated for 7 days with Dox diet (2g/kg) prior to euthanasia and mammary gland dissection. The number 4 mammary gland pairs were dissected, fixed in 4% PFA, and embedded in paraffin. Five µm sections were cut for histological and immunostaining analysis. The tissues were stained by standard H&E, Masson's Trichrome Stain kit (Sigma, HT15-1KT), or immunostaining to study specific proteins. For immunostaining, the tissues were deparaffinized in xylene and rehydrated through ethanols of decreasing concentration. Antigen retrieval involved microwaving the slides in 10 mM Sodium Citrate, pH 6 for 20 minutes. Five percent BSA plus 0.5% tween-20 in PBS was used as a block and diluent for antibodies. Sections were incubated overnight with primary antibodies and for one hour with secondary antibodies. For immunohistochemistry, Elite ABC Reagent (Vector Laboratories, PK7100) and DAB (SK-4100) were used to develop the immunoperoxidase staining and the slides were counterstained with hematoxylin (Fischer, CS400-1D). For immunofluorescence, the nuclei were stained with DAPI. Primary antibodies included: Ki67 1∶5000 (Abcam, ab15580); LOX 1∶250 no antigen retrieval (Abcam, ab31238). Secondary antibodies consisted of anti-rabbit Alexa Fluor 555 (Invitrogen, A21428) and biotinylated anti-rabbit (Vector Laboratories, BA1000). To quantify stromal thickness on H&E sections, the thickest regions of the stroma were measured at the tip and neck of longitudinally sectioned TEBs using ImageJ software. The area of positive staining through Masson's trichrome and LOX immunofluorescence was scored on a 0–3 scale with 0 being negative for staining. TEBs were analyzed from 5 mice per genotype.

### Contraction Assay

MEC contractility was analyzed using Cell Contraction Assays (Cell Biolabs, Inc., CBA201) conducted according to the manufacturer's instructions. Growth media (MEGM BulletKit) with 2 µg/ml Dox was added after the gels solidified and fresh media was added when the gels were released. Quantification of gel contraction was measured using images of the gels captured immediately after their release and 24 hours after release. Gel diameter was determined by averaging the diameter in 2 planes perpendicular to each other. The imaging and quantification was done with a Zeiss Axioimager A1 epifluorescence microscope. The assay was conducted in triplicate and the data represent the average of 3 independent experiments.

### RNA isolation and quantitative RT-PCR

RNA was isolated from control and p190B-associated fibroblasts and wildtype fibroblasts from conditioned media and co-culture experiments using Trizol (Invitrogen, 15596-018) and RNeasy RNA purification columns (Qiagen, 74104) according to the manufacturer's recommendations. One µg of RNA was converted to cDNA using the RT^2^ First Strand Kit (Qiagen, 330401). Control and p190B-associated fibroblast cDNA was amplified using RT^2^ Profiler PCR Array Mouse Extracellular Matrix and Adhesion Molecules (PAMM-013A) per manufacturer's instructions. Data were analyzed using the web-based software RT^2^ Profiler PCR Array Data Analysis from SABiosciences. Wildtype fibroblast cDNA from conditioned media and co-culture experiments was amplified using RT^2^ SYBR Green qPCR Master Mix (Qiagen, 330522), the StepOnePlus Real-Time PCR System (Applied Biosystems), and the following primers: *Col1a1* primers: 5′-GCT CCT CTT AGG GGC CAC T-3′ and 5′-CCA CGT CTC ACC ATT GGG G-3′; *Fn1* primers: 5′-TTC AAG TGT GAT CCC CAT GAA G-3′ and 5′-CAG GTC TAC GGC AGT TGT CA-3′; *Lama1* primers: 5′-CAG CGC CAA TGC TAC CTG T-3′ and 5′-GGA TTC GTA CTG TTA CCG TCA CA-3′; *Ctgf* primers: 5′-GGG CCT CTT CTG CGA TTT C-3′ and 5′-ATC CAG GCA AGT GCA TTG GTA-3′; and *Gapdh* primers: 5′-CCA ATH TGT CCG TCG TGG ATC-3′ and 5′-GTT GAA GTC GCA GGA GAC AAC-3′. The reaction conditions were as follows: 95°C for 10 minutes then 40 cycles of 95°C for 15 seconds and 60°C for 1 minute followed by a melting curve. *Col1a1*, *Fn1*, *Lama1*, and *Ctgf* mRNA levels were normalized to *GAPDH* mRNA levels and the data were analyzed using comparative C_T_. Each experiment was conducted in triplicate and the data represent 2–3 independent experiments each consisting of 3–4 animals per genotype pooled.

### Western blotting

Lysates were made from pulverized whole glands, isolated organoids and fibroblasts, or wildtype fibroblasts isolated from conditioned media and co-culture experiments with RIPA buffer containing a protease inhibitor cocktail (Thermo Scientific, 1862209) and the phosphatase inhibitors sodium fluoride (s1504) and sodium orthovanadate (S6508). After a 10 minute incubation on ice, the lysates were centrifuged at 13,000 rpm at 4°C for 10 minutes. A BCA assay (Pierce, 23225) was used to determine total protein concentration of the supernatant. Lysates were electrophoresed on 6%, 10% or 15% SDS polyacrylamide gels and transferred to PVDF membranes. After blocking in 5% milk TBST for 1 hour at room temperature, the membranes were incubated overnight at 4°C with primary antibodies diluted 1∶1000 in 5% milk TBST or 5% BSA TBST. Primary antibodies used for immunoblotting included: p190B (BD, 611612), vimentin (Santa Cruz, sc-73262), α smooth muscle actin (Sigma, A5228), β-tubulin (Sigma, T5201), β-actin (Sigma, A5441), lysyl oxidase (Abcam, ab31238), pMLC2 Ser19 (Cell Signaling, 3671), pERK 1/2 Thr202/Tyr308 (Cell Signaling, 4370), ERK 1/2 (Cell Signaling, 4695), E-cadherin (BD, 610181), pSMAD2 Ser465/467 (Cell Signaling, 3108), SMAD2 (Cell Signaling, 5339), CTGF (Abcam, ab5097), collagen (Abcam, ab34710), fibronectin (Santa Cruz, sc-9068), laminin (Abcam, ab11575), TGF-β (Cell Signaling, 3711) Keratin 14 (Convance, PRB-155P), and fibroblast specific protein 1 (FSP1) (Abcam, ab27957). Secondary antibodies were diluted 1∶1000 or 1∶5000 in 5% milk TBST and incubated for 1 hour at room temperature. Secondary antibodies included: HRP conjugated anti-mouse or anti-rabbit (Jackson ImmunoResearch, 115-035-003, 111-035-003). All washes were done with TBST. Immunoblots were developed with West Pico (Pierce, 34077), West Femto (34095), or ECL Advance Solution (GE Healthcare, RPN2135). Data were normalized to β-actin or β-tubulin for cell lysates and ponceau for conditioned media and represent fold change compared to control samples. ImageJ software was used to analyze the developed blots. The lysates and conditioned media represented protein pooled from 3–5 mice per genotype.

### Luciferase assays

Lysates were made from organoids and fibroblasts pooled from 5 animals per genotype with passive lysis buffer (Promega, E1941). After a 10 minute incubation on ice, the lysates were centrifuged at 13,000 rpm at 4°C for 10 minutes, and a BCA assay (Pierce, 23225) was used to determine total protein concentration of the supernatant. Lysates were allowed to warm to room temperature before luciferase substrate (Promega, E148) was added. A GloMax 20/20 Luminometer (Promega) was used to read luciferase activity, which was normalized to total protein levels of the lysates.

### Statistical analysis

Fisher's exact test was used to analyze the statistical significance of categorical data. Unpaired Student's T-test was used to analyze all other data. P values of ≤0.05 were considered statistically significant. Error bars represent the standard error of the mean.

## Results

### P190B overexpression in the developing mammary epithelium is associated with increased stromal deposition in vivo

**Figure 1:**
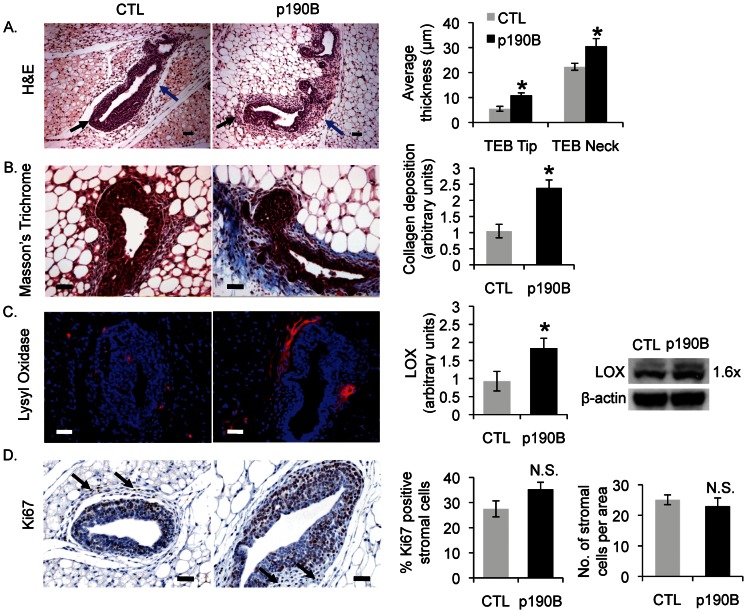
P190B overexpression in the developing mammary epithelium is associated with stromal alterations. A. Representative images of H&E stained control and p190B overexpressing TEBs with quantification of stromal thickness demonstrating increased thickness of the stroma at the neck (blue arrows) (p = 0.04) and tip (black arrows) (p = 0.005) regions of p190B overexpressing TEBs compared to control TEBs (n = 5 mice/genotype with at least 5 TEBs analyzed per animal). Magnification = 200X. B. Representative images of Masson's Trichrome staining (collagen, blue) and the corresponding quantification demonstrating increased collagen deposition around p190B overexpressing TEBs (n = 5 mice/genotype with at least 3 TEBs analyzed per animal), *p = 0.003. C. Representative images of LOX immunofluorescence (red), corresponding quantification, and an immunoblot of LOX on lysates from control and p190B overexpressing whole glands showing increased LOX expression in p190B overexpressing glands (immunofluorescence: n = 4–5 mice/genotype with at least 4 TEBs per animal; immunoblot: equal amounts of protein pooled from n = 5 mice/genotype), *p = 0.05. D. Representative images of Ki67 immunohistochemistry with corresponding quantification of stromal cell proliferation (arrows indicate positive cells in the stroma) and cell number with respect to area demonstrates no significant difference in proliferation between control and p190B overexpressing TEBs (n = 5 mice/genotype with at least 3 TEBs per animal) as well as no difference between the number of stromal cells per stromal area (n = 4 mice/genotype with at least 3 TEBs per animal). Magnification for panels B-D = 400X and all size bars = 20 µm.

We previously reported that ECM deposition appeared to be increased in association with dysmorphic, hyperbranched terminal end buds (TEBs) in the p190B overexpressing mammary glands [18]. To confirm and extend these results, we measured and quantified stromal thickness and collagen deposition associated with the TEBs of p190B overexpressing mice compared to Dox-treated MMTV-rtTA (control) mice. Condensed stroma is typically found adjacent to the neck region of the TEB, whereas the tip of the TEB is usually surrounded by only a thin basement membrane [22]. In comparison to control TEBs, the stroma surrounding p190B overexpressing TEBs was significantly thicker at the neck regions (22.3+/−1.39 vs. 30.6+/−3.1 µm, p = 0.04) as well as at the tips of the TEBs (5.51+/−0.98 vs. 10.9+/−1.0 µm, p = 0.005) (Figure 1A). Collagen accumulation was analyzed using Masson's Trichrome stain on mammary gland tissue sections, and the stroma adjacent to p190B overexpressing TEBs exhibited significantly more collagen deposition compared to control TEBs (Figure 1B). In addition, immunostaining for lysyl oxidase (LOX), an enzyme that cross-links collagen making it more rigid [23], showed that LOX is also increased in the stroma associated with p190B overexpressing TEBs (Figure 1C). This was further supported by Western analysis of whole mammary gland lysates, which showed a 1.6-fold increase in expression of the 50 kDa pro form of LOX. Collectively, these data indicate that p190B overexpression in the mammary gland leads to increased ECM deposition and potentially reorganization of the ECM.

Increased stromal deposition could result from an increase in the number of fibroblasts associated with the TEBs and/or elevated fibroblast activity. To evaluate this, tissue sections were immunostained with the proliferation marker Ki67, and the percentage of Ki67 positive stromal cells associated with the TEBs was quantified. No significant differences in Ki67 positivity were detected in the stromal cells associated with p190B overexpressing TEBs compared with control TEBs (Figure 1D). In addition, quantification of the number of stromal cells with respect to stromal area was not altered in the p190B overexpressing TEBs (Figure 1E). These data indicate that p190B overexpression does not lead to a significant expansion or recruitment of stromal cells, suggesting that the increased stromal deposition is due to elevated fibroblast activity.

### Fibroblasts isolated from p190B overexpressing mammary glands display increased ECM production and activation of TGF-β signaling

**Figure 2:**
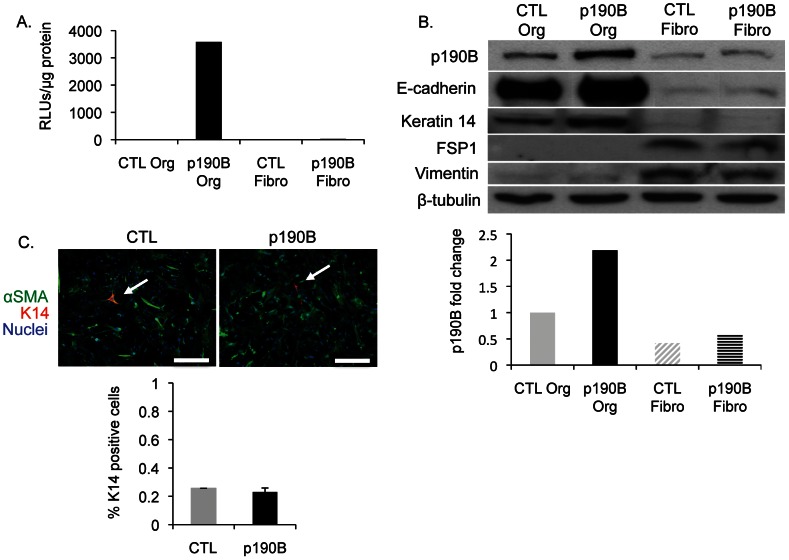
P190B overexpression is confined to the epithelium in p190B overexpressing mammary glands. A. Luciferase assay showing that luciferase activity is only detected in the p190B organoids indicating that transgene expression is confined to the epithelial cell compartment of p190B overexpressing mammary glands (n = 5 mice/genotype pooled). B. Immunoblot with corresponding densitometry demonstrating approximately a 2-fold increase in p190B expression in p190B organoids compared to control organoids and no significant difference in p190B levels between fibroblasts from control and p190B overexpressing glands. E-cadherin and keratin 14 (K14), an epithelial cell and myoepithelial cell marker, respectively, and fibroblast specific protein 1 (FSP1) and vimentin, mesenchymal markers, demonstrate the efficacy of the method used to separate organoids and fibroblasts (n = 5 mice/genotype pooled). C. Immunostaining of the fibroblast population for α smooth muscle actin (αSMA) and K14 and the corresponding quantification of K14 positive cells confirming very little myoepithelial contamination of the isolated fibroblasts (n>500 cells/genotype).

We next wanted to investigate whether increased stromal deposition was due to increased fibroblast activity and ECM secretion in the p190B overexpressing mammary glands. Before examining this, we first confirmed that the p190B transgene was overexpressed in the mammary epithelium, but not in the mammary gland fibroblasts. Inducible overexpression of the p190B transgene within the mouse mammary gland is under the control of the reverse tetracycline transactivator (rtTA) driven by the MMTV promoter [21], which allows for induction of p190B overexpression in the MECs upon doxycycline (Dox) treatment. The transgene construct also contains an IRES-luciferase reporter allowing for rapid confirmation of transgene expression. Analysis of luciferase activity showed that the p190B transgene was expressed in organoids (mammary epithelial fragments containing luminal and myoepithelial cells) isolated from p190B overexpressing mammary glands, but not in organoids isolated from control mammary glands (Figure 2A). As expected, the fibroblasts from both p190B and control mammary glands were negative for luciferase activity. Western analysis showed that p190B protein levels were increased 2-fold in organoids isolated from p190B overexpressing mammary glands compared to control organoids, whereas p190B protein levels were comparable in the fibroblast populations (Figure 2B). Western blotting for E-cadherin and K14, epithelial markers, and vimentin and FSP-1, fibroblast markers, confirmed the identity of the isolated cells and showed that there was a low and equivalent level of contamination of the isolated cells with the other cell type. In addition, immunostaining and quantification of the percentage of cells expressing the myoepithelial marker K14 and alpha smooth muscle actin (αSMA), a marker of myoepithelial cells and activated fibroblasts, confirmed that there was little contamination of the isolated fibroblasts with myoepithelial cells and that it was equivalent between the p190B-associated and control fibroblast populations (Figure 2C). Thus, our methods for isolation of fibroblasts and MECs yield relatively pure populations of each cell type. Furthermore, these data confirm that the p190B transgene is overexpressed in the MECs, and as expected, it is not expressed in the fibroblasts.

**Figure 3:**
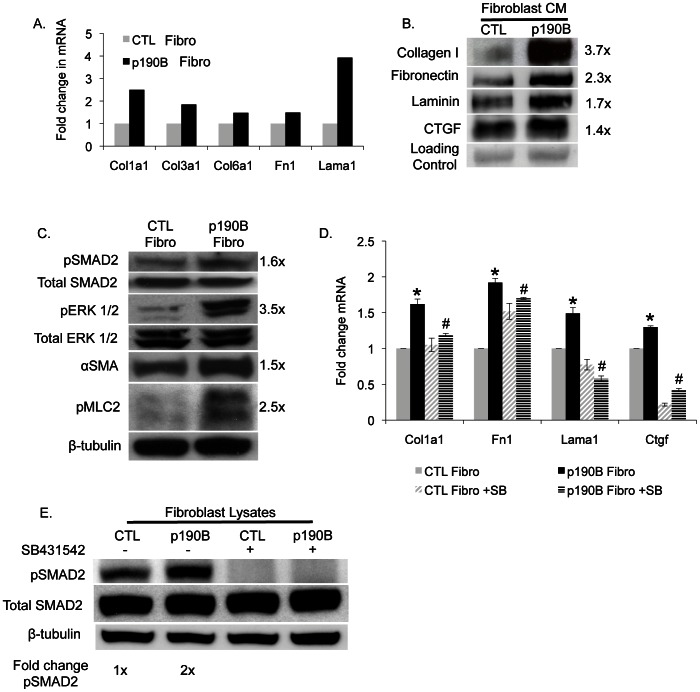
P190B-associated fibroblasts display elevated ECM production that depends on TGF-β signaling. A. qRT-PCR Super Array for ECM proteins showing increased collagen (*Col1a1*, *Col3a1*, *Col6a1*), fibronectin (*Fn1*), and laminin alpha 1 (*Lama1*) mRNA expression in fibroblasts associated with p190B overexpressing MECs (n = 5 mice/genotype pooled) compared to fibroblasts associated with control MECs. B. Immunoblot of conditioned medium (CM) from CTL and p190B-associated fibroblasts showing increased secretion of collagen, fibronectin, laminin, and CTGF by p190B-associated fibroblasts. Values to the right of the blots represent fold change with respect to control CM and were normalized to ponceau loading control (CM from n = 3 mice/genotype pooled). C. Immunoblots performed on fibroblasts isolated from control and p190B overexpressing glands showing increased pSMAD2, pERK1/2, αSMA, and pMLC in p190B-associated fibroblasts. Values to the right of the blots represent fold change with respect to control fibroblasts and were normalized to β-tubulin (n = 5 mice/genotype pooled). D. qRT-PCR on fibroblasts isolated from control and p190B overexpressing glands and treated with DMSO or 5 µM SB431542 (SB) showing that TGFβ receptor inhibition blocks the increased expression of *Col1a1*, *Lama1*, and *Ctgf* mRNA in the p190B-associated fibroblasts (n = 4 mice/genotype pooled), *p≤0.001 (p190B vs. control), ^#^p≤0.04 (p190B vs. p190B+SB). E. Immunoblot of lysates prepared from fibroblasts isolated from control and p190B overexpressing glands and treated in culture with DMSO or 5 µM SB431542 demonstrating that SB431542 effectively blocks TGFβ signaling through SMAD2. Values were normalized to β-tubulin (n = 4 mice/genotyped pooled).

Increased ECM production and expression of αSMA are features of activated fibroblasts [24]. In order to determine if epithelial overexpression of p190B altered the activity of adjacent fibroblasts, ECM production by p190B-associated and control fibroblasts was compared. Fibroblasts from p190B overexpressing or control mammary glands were isolated, and ECM mRNA expression levels were analyzed using a qRT-PCR Super Array for ECM genes. Elevated mRNA expression levels of the ECM components collagen type 1 alpha1 (*Col1a1*), *Col3a1*, *Col6a1*, fibronectin (*Fn1*), and laminin alpha 1 (*Lama1*) were detected in the p190B-associated fibroblasts as compared to fibroblasts isolated from control mammary glands (Figure 3A and Table S1).

To determine if ECM protein expression levels were also increased, fibroblasts isolated from p190B overexpressing and control mammary glands were cultured, and conditioned medium (CM) was prepared from these cultures. Western blotting revealed a 3.7-fold increase in type I collagen, 2.3-fold increase in fibronectin, and 1.7-fold increase in laminin secretion by the p190B-associated fibroblasts as compared to control fibroblasts (Figure 3B). In addition, secretion of connective tissue growth factor (CTGF), a potent regulator of ECM production in fibroblasts [25], [26], was elevated 1.4-fold in p190B-associated fibroblasts compared to control fibroblasts. These data demonstrate that ECM gene expression and protein secretion are significantly increased in p190B-associated fibroblasts.

The TGF-β signaling network is a well-known mediator of fibroblast activation, ECM production, and tissue fibrosis [27]. We therefore examined whether this signaling pathway was differentially activated in the p190B-associated fibroblasts compared to control fibroblasts. Western analysis of protein lysates prepared from fibroblasts that were freshly isolated from mammary glands showed that phosphorylation of SMAD2, a key downstream effector of TGF-β signaling [28], [29], was elevated 1.6-fold in p190B-associated fibroblasts (Figure 3C). ERK activation cooperates with TGF-β signaling to induce ECM gene expression [30], [31], and Western analysis showed that phosphorylation of ERK was markedly increased by 3.5-fold in the p190B-associated fibroblasts. Increased actomyosin contractility is a feature of activated fibroblasts, and αSMA, a downstream target of TGF-β signaling [32], was upregulated 1.5-fold and phosphorylation of myosin light chain 2 (MLC2) was increased 2.5-fold in fibroblasts isolated from p190B overexpressing mammary glands compared to control mammary glands.

To determine if TGF-β signaling is necessary in the p190B-associated fibroblasts for increased ECM production, p190B-associated and control fibroblasts were isolated from p190B overexpressing and control mammary glands, respectively, plated in culture, and treated with a TGF-β receptor inhibitor SB431542 or DMSO vehicle control. After 48 hours, mRNA was prepared from the fibroblasts and qRT-PCR was done for *Col1a1*, *Fn1*, *Lama1*, and *Ctgf*. These results showed that inhibition of TGF-β signaling significantly reduced the expression of *Col1a1*, *Lama1*, and *Ctgf* (Figure 3D). In addition, Western blotting confirmed that SB431542 treatment effectively blocked TGF-β signaling as phosphorylation of SMAD2 was undetectable in both p190B-associated and control fibroblasts after treatment (Figure 3E). Collectively, these data suggest that activation of p190B-associated fibroblasts is likely due to increased TGF-β signaling.

### Paracrine signals from the p190B overexpressing mammary epithelium induce fibroblast activation

**Figure 4:**
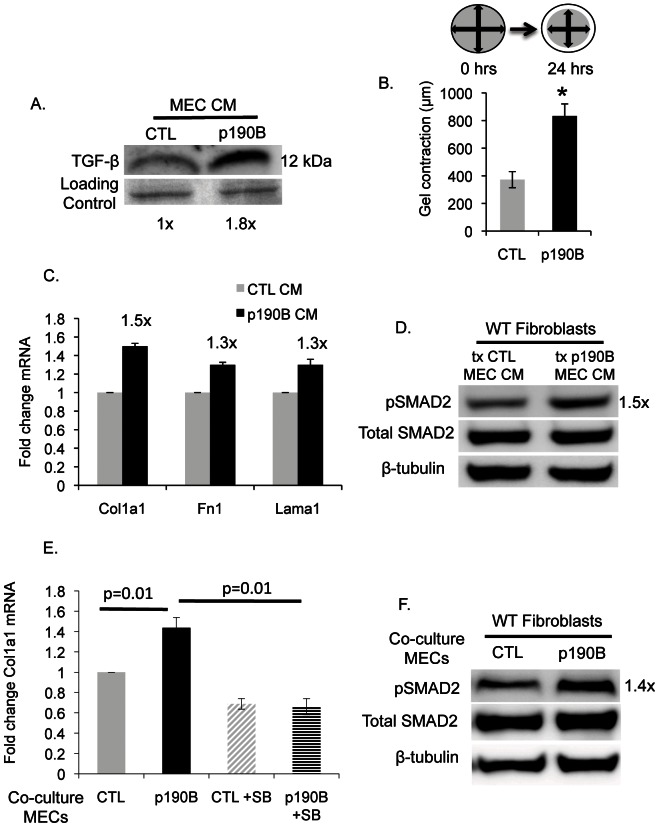
Paracrine signals from p190B overexpressing MECs upregulate TGF-β dependent ECM mRNA expression in fibroblasts. A. Representative immunoblot of MEC CM showing increased levels of active TGF-β. The value to the right of the blot represents fold change with respect to control MEC CM and is normalized to ponceau loading control. (CM from n = 4 mice/genotype pooled). B. A schematic representing the contraction assay is shown. Quantification of contraction assay on CTL and p190B overexpressing MECs demonstrating increased contractility of p190B overexpressing MECs compared to control MECs. Data represents the average of 3 independent experiments, *p = 0.006. C. qRT-PCR on wildtype fibroblasts treated with CM from CTL or p190B overexpressing MECs showing that p190B CM induces increased gene expression of *Col1a1*, *Fn1*, and *Lama1* in wildtype fibroblasts (CM from n = 4 mice/genotype pooled). D. Immunoblot on wildtype fibroblasts treated with CTL or p190B CM demonstrating that CM from p190B overexpressing MECs increased SMAD2 phosphorylation in wildtype fibroblasts compared to CM from CTL MECs. The value to the right of the blot represents fold change with respect to treatment with CTL CM and is normalized to β-tubulin (wildtype fibroblasts treated with CM from n = 4 mice/genotype pooled). E. qRT-PCR on wildtype fibroblasts that were co-cultured with CTL or p190B overexpressing MECs showing that co-culture with p190B overexpressing MECs increased gene expression of *Col1a1* in wildtype fibroblasts compared to co-culture with CTL MECs. Additionally, treatment of co-cultures with 5 µM SB431542 prevents the increased *Col1a1* mRNA expression in wildtype fibroblasts co-cultured with p190B overexpressing MECs. Data represent the average of 3 independent experiments with MECs from n = 3–4 mice/genotype pooled per experiment, *p = 0.01. F. Immunoblot on wildtype fibroblasts that were co-cultured with CTL or p190B overexpressing MECs demonstrating that co-culture with p190B overexpressing MECs increased SMAD2 phosphorylation in wildtype fibroblasts. Value to the right of the blot represents fold change with respect to wildtype fibroblasts co-cultured with CTL MECs and is normalized to β-tubulin (wildtype fibroblasts in co-culture with n = 4 mice/genotype pooled).

TGF-β, an important regulator of mammary gland branching morphogenesis, is expressed by the mammary epithelium and acts in an epithelial autonomous manner to regulate MEC proliferation and branching in the developing postnatal mammary gland [33], [34], [35]. In addition, TGF-β ligands are also produced by fibroblasts where they can act in an autocrine and paracrine fashion to affect fibroblast activity [36], [37], [38]. We were interested in determining whether the elevated TGF-β signaling detected in the p190B-associated fibroblasts might be mediated by the epithelium. To test this, MECs were isolated from p190B overexpressing and control mammary glands, plated in culture, and CM was collected. Active TGF-β is a 25 kDa homodimer, which is detected as a 12 kDa protein under reducing conditions by Western blotting. Intriguingly, a 1.8-fold increase in active TGF-β was detected in the CM from p190B overexpressing MECs compared to control MECs (Figure 4A).

Activation of fibroblasts by TGF-β also requires increased mechanical tension [36], [37], and p190B regulates the cytoskeleton and intracellular tension [16]. We therefore investigated whether p190B overexpressing MECs might have increased cytoskeletal contractility that could create an environment of increased mechanical tension. Interestingly, p190B overexpressing MECs displayed a 2-fold increase in contractility in a collagen gel-based contraction assay when compared to control MECs (Figure 4B). These results suggest that p190B overexpressing MECs display increased production of active TGF-β and increased mechanical tension, both of which contribute to fibroblast activation.

Next, to investigate whether paracrine signals from the p190B overexpressing MECs are involved in activation of the adjacent fibroblasts, CM from p190B overexpressing or control MECs was transferred onto fibroblasts isolated from the mammary glands of wildtype FVB mice. After 48 hours, mRNA was isolated from the fibroblasts and qRT-PCR for *Col1a1*, *Fn1*, and *Lama1* was done. Fibroblasts treated with CM from p190B overexpressing MECs showed increases in *Col1a1, Fn1,* and *Lama1* mRNA expression that were similar to the levels detected in cultured p190B-associated fibroblasts (as shown in Figure 3D) when compared to fibroblasts treated with CM from control MECs (Figure 4C). Furthermore, Western analysis revealed a 1.5-fold increase in phosphorylated SMAD2 in the fibroblasts treated with CM from p190B overexpressing MECs, suggesting that the p190B CM increased ECM mRNA expression via activation of TGF-β signaling (Figure 4D). These results are consistent with the idea that elevated levels of active TGF-β are produced by the p190B overexpressing MECs and function in a paracrine manner to stimulate fibroblast activity.

To further explore the mechanisms by which p190B overexpressing MECs induce fibroblast activation, we turned to a MEC-fibroblast co-culture model. This model allowed for extended culture times as compared to CM treatment, which limited the duration that the fibroblasts could be cultured because the CM was not optimal for fibroblast growth. To establish MEC-fibroblast co-cultures, p190B overexpressing MECs or control MECs were plated at a 2.5∶1 ratio with fibroblasts isolated from wildtype FVB mammary glands and cultured for 72–96 hours. Fibroblasts were recovered from the cultures, mRNA was isolated, and qRT-PCR for *Col1a1* mRNA expression levels was done. This analysis demonstrated 1.4-fold and 1.8-fold increases in *Col1a1* mRNA expression after 72 and 96 hours, respectively, in fibroblasts co-cultured with p190B overexpressing MECs as compared to control MECs (Figure 4E and data not shown). In addition, phosphorylated SMAD2 was increased in fibroblasts co-cultured with p190B overexpressing MECs, indicating that the co-culture conditions recapitulate the CM experiments (Figure 4F). We next determined whether TGF-β signaling is required for the effects of p190B overexpressing MECs on fibroblast activation as measured by increased *Col1a1* mRNA expression. The co-culture experiments were repeated in the presence of SB431542, which effectively blocked signaling through the TGF-β pathway because phosphorylated levels of SMAD2 were undetectable after SB431542 treatment (data not shown). SB431542 also prevented the increase in *Col1a1* mRNA expression detected in the fibroblasts co-cultured with the p190B overexpressing MECs (Figure 4E). Taken together, these data demonstrate that paracrine signals from the p190B overexpressing MECs induce increased ECM production by associated fibroblasts likely via a TGF-β dependent mechanism.

## Discussion

Proper regulation of ECM deposition and organization is important for normal mammary gland morphogenesis [39], and aberrant ECM deposition and rigidity promote abnormal MEC morphogenesis and facilitate the development and progression of breast cancer [12], [13], [40], [41]. However, the mechanisms by which the mammary epithelium affects stromal ECM deposition during normal and neoplastic mammary gland development are not well understood. Here we show that a low level of p190B RhoGAP overexpression in the mammary epithelium induces stromal activation characterized by increased fibroblast activity and ECM deposition. Furthermore, our data indicate that p190B overexpressing MECs produce elevated levels of active TGF-β and that paracrine signals from p190B overexpressing MECs induce ECM gene expression in fibroblasts via activation of TGF-β signaling. Although induction of the p190B transgene only increases p190B expression 2-fold over endogenous levels in control mammary glands, this is nonetheless an overexpression model. It will be important to investigate the effects of p190B deficiency within the mammary epithelium on stromal activation to determine if this is an essential physiological function of p190B in the developing mammary gland.

TGF-β signaling is a well-known inducer of fibroblast activation [29], and our data demonstrate that this pathway is upregulated in the p190B-associated fibroblasts. Studies show that TGF-β signaling during fibroblast activation occurs in a cell autonomous fashion [27]. However, our results suggest that paracrine signals originating from the p190B overexpressing MECs increase TGF-β dependent ECM production in the p190B-associated fibroblasts. It has also been demonstrated that mechanical tension derived from the ECM and intracellular contractility cooperate with TGF-β to activate fibroblasts [36], [37], [38], [42]. The increased contractility of p190B overexpressing MECs, increased ECM deposition, and elevated phosphorylated MLC2 and αSMA levels in the p190B-associated fibroblasts are indicative of increased mechanical tension within the p190B overexpressing TEBs and adjacent stroma. These altered mechanical properties may also contribute to the activation of p190B-associated fibroblasts, although future experiments will be required to directly demonstrate the importance of altered mechanical forces in this process. In addition, although our conditioned medium and co-culture experiments show that p190B overexpressing MECs can induce TGF-β dependent fibroblast activation, it is possible that this may lead to increased autocrine signaling in the fibroblasts to further promote their activation. Secretion of CTGF, a potent regulator of ECM production that cooperates with TGF-β to regulate fibroblast activity [25], [26], was upregulated in the p190B-associated fibroblasts, suggesting that an autocrine loop may also be active.

Proteolytic and integrin-mediated, cytoskeletal-dependent mechanisms have been shown to activate latent TGF-β [43]. Our data demonstrating that p190B overexpressing MECs display elevated contractility suggests that an integrin/cytoskeletal-mediated mechanism may contribute to the increase in TGF-β activation by the p190B overexpressing MECs. However, future studies will be required to define the mechanisms by which p190B overexpression in MECs leads to increased activation of TGF-β.

An important question that remains to be answered is how the altered stromal environment affects the developing mammary epithelium in the p190B overexpressing mammary glands. P190B overexpressing TEBs display increased proliferation, dysmorphic morphologies, and hyperbranching in association with fibroblast activation, increased ECM deposition, and potentially reorganization of the ECM by LOX. Increased ECM deposition and rigidity are known to disrupt MEC morphogenesis [3], [12]. However, it is currently unclear whether the aberrant TEB morphogenesis that occurs in p190B overexpressing mammary glands is MEC autonomous or whether mechanical cues resulting from the altered stroma contribute to these phenotypes. Activated fibroblasts also produce growth factors and cytokines that impact MEC morphogenesis [44]. Thus, it is possible that the p190B-associated fibroblasts secrete factors that impact proliferation and branching of the adjacent epithelium. Future studies will be required to define the relative contributions of mechanical and soluble signals generated by the p190B-associated fibroblasts as well as epithelial autonomous effects to the p190B overexpressing TEB phenotypes.

Rho GTPase expression and activity levels are elevated in human breast tumors [7], [8], and aberrant Rho signaling affects transformation, tumor cell proliferation, and migration and invasion, all of which are important for tumorigenesis and metastasis [9]. Our data as well as data from reported studies [14] suggest that aberrant Rho signaling in the epithelial compartment may also play an important role in altering interactions between the epithelium and stroma, leading to fibroblast activation and increased ECM deposition. Thus, stromal activation resulting from aberrant Rho signaling in the epithelial compartment may be another mechanism by which Rho signaling contributes to tumorigenesis.

## Supporting Information

Table S1 **qRT-PCR Super Array analysis shows changes in ECM and adhesion molecule gene expression between p190B-associated and control fibroblasts.** The RT^2^ Profiler PCR Array Mouse Extracellular Matrix and Adhesion Molecules platform was used to compare changes in gene expression between the two fibroblast populations. Gene symbol, description, and fold change in expression for the p190B-associated fibroblasts compared to control (CTL) fibroblasts are shown.
(PDF)
