# Supplementary material for: P190B RhoGAP Overexpression in the Developing Mammary Epithelium Induces TGFβ-dependent Fibroblast Activation
Source: PLoS One. 2013 May 22;8(5):e65105. doi: 10.1371/journal.pone.0065105 (PMC3661508; doi:10.1371/journal.pone.0065105)
Supplement: Table S1 — qRT-PCR Super Array analysis shows changes in ECM and adhesion molecule gene expression between p190B-associated and control fibroblasts. The RT2 Profiler PCR Array Mouse Extracellular Matrix and Adhesion Molecules platform was used to compare changes in gene expression between the two fibroblast populations. Gene symbol, description, and fold change in expression for the p190B-associated fibroblasts compared to control (CTL) fibroblasts are shown. (PDF) [file pone.0065105.s001.pdf]

Table S1

RT-qPCR Superarray on CTL and p190B-Associated Fibroblasts for Extracellular Matrix and Adhesion Proteins

Well	Symbol	Fold Change with Respect to CTL	Description
A01	Adamts1	0.9971	A disintegrin-like and metallopeptidase (reprolysin type) with thrombospondin type 1 motif, 1
A02	Adamts2	1.5275	A disintegrin-like and metallopeptidase (reprolysin type) with thrombospondin type 1 motif, 2
A03	Adamts5	1.1482	A disintegrin-like and metallopeptidase (reprolysin type) with thrombospondin type 1 motif, 5 (aggrecanase-2)
A04	Adamts8	1.2799	A disintegrin-like and metallopeptidase (reprolysin type) with thrombospondin type 1 motif, 8
A05	Ctnna1	1.0117	Catenin (cadherin associated protein), alpha 1
A06	Ctnna2	0.1672	Catenin (cadherin associated protein), alpha 2
A07	Ctnnb1	1.0559	Catenin (cadherin associated protein), beta 1
A08	Cd44	1.3295	CD44 antigen
A09	Cdh1	0.994	Cadherin 1
A10	Cdh2	0.7226	Cadherin 2
A11	Cdh3	0.9111	Cadherin 3
A12	Cdh4	0.305	Cadherin 4
B01	Cntn1	0.6286	Contactin 1
B02	Col1a1	2.5167	Collagen, type I, alpha 1
B03	Col2a1	0.8712	Collagen, type II, alpha 1
B04	Col3a1	1.8519	Collagen, type III, alpha 1
B05	Col4a1	1.1992	Collagen, type IV, alpha 1
B06	Col4a2	1.2861	Collagen, type IV, alpha 2
B07	Col4a3	1.2083	Collagen, type IV, alpha 3
B08	Col5a1	1.3793	Collagen, type V, alpha 1
B09	Col6a1	1.4815	Collagen, type VI, alpha 1
B10	Vcan	1.2025	Versican
B11	Ctgf	1.5965	Connective tissue growth factor
B12	Ecm1	1.1302	Extracellular matrix protein 1

RT-qPCR Superarray on CTL and p190B-Associated Fibroblasts for Extracellular Matrix and Adhesion Proteins

Well	Symbol	Fold Change with Respect to CTL	Description
C01	Emilin1	1.301	Elastin microfibril interfacer 1
C02	Entpd1	0.9732	Ectonucleoside triphosphate diphosphohydrolase 1
C03	Fbln1	1.2551	Fibulin 1
C04	Fn1	1.4903	Fibronectin 1
C05	Hapln1	0.6506	Hyaluronan and proteoglycan link protein 1
C06	Hc	1.5636	Hemolytic complement
C07	Icam1	0.8552	Intercellular adhesion molecule 1
C08	Itga2	0.718	Integrin alpha 2
C09	Itga3	0.9812	Integrin alpha 3
C10	Itga4	0.2093	Integrin alpha 4
C11	Itga5	0.7215	Integrin alpha 5 (fibronectin receptor alpha)
C12	Itgae	1.3418	Integrin alpha E, epithelial-associated
D01	Itgal	0.9913	Integrin alpha L
D02	Itgam	1.014	Integrin alpha M
D03	Itgav	1.1476	Integrin alpha V
D04	Itgax	1.593	Integrin alpha X
D05	Itgb1	1.3743	Integrin beta 1 (fibronectin receptor beta)
D06	Itgb2	1.0899	Integrin beta 2
D07	Itgb3	0.9059	Integrin beta 3
D08	Itgb4	1.4523	Integrin beta 4
D09	Lama1	3.9334	Laminin, alpha 1
D10	Lama2	0.9873	Laminin, alpha 2
D11	Lama3	1.31	Laminin, alpha 3
D12	Lamb2	1.2149	Laminin, beta 2

RT-qPCR Superarray on CTL and p190B-Associated Fibroblasts for Extracellular Matrix and Adhesion Proteins

Well	Symbol	Fold Change with Respect to CTL	Description
E01	Lamb3	0.9316	Laminin, beta 3
E02	Lamc1	0.9103	Laminin, gamma 1
E03	Mmp10	0.9358	Matrix metallopeptidase 10
E04	Mmp11	0.6334	Matrix metallopeptidase 11
E05	Mmp12	1.0761	Matrix metallopeptidase 12
E06	Mmp13	1.0042	Matrix metallopeptidase 13
E07	Mmp14	1.2634	Matrix metallopeptidase 14 (membrane-inserted)
E08	Mmp15	0.7696	Matrix metallopeptidase 15
E09	Mmp1a	0.5898	Matrix metallopeptidase 1a (interstitial collagenase)
E10	Mmp2	1.3515	Matrix metallopeptidase 2
E11	Mmp3	1.1641	Matrix metallopeptidase 3
E12	Mmp7	0.3864	Matrix metallopeptidase 7
F01	Mmp8	1.3408	Matrix metallopeptidase 8
F02	Mmp9	0.5284	Matrix metallopeptidase 9
F03	Ncam1	1.7364	Neural cell adhesion molecule 1
F04	Ncam2	1.2701	Neural cell adhesion molecule 2
F05	Pecam1	0.5578	Platelet/endothelial cell adhesion molecule 1
F06	Postn	1.4438	Periostin, osteoblast specific factor
F07	Sele	0.7194	Selectin, endothelial cell
F08	Sell	0.4661	Selectin, lymphocyte
F09	Selp	0.721	Selectin, platelet
F10	Sgce	1.228	Sarcoglycan, epsilon
F11	Sparc	1.4152	Secreted acidic cysteine rich glycoprotein
F12	Spock1	1.0089	Sparc/osteonectin, cwcv and kazal-like domains proteoglycan 1

RT-qPCR Superarray on CTL and p190B-Associated Fibroblasts for Extracellular Matrix and Adhesion Proteins

Well	Symbol	Fold Change with Respect to CTL	Description
G01	Spp1	0.6483	Secreted phosphoprotein 1
G02	Syt1	15.5934	Synaptotagmin I
G03	Tgfbi	0.9465	Transforming growth factor, beta induced
G04	Thbs1	1.2409	Thrombospondin 1
G05	Thbs2	1.0308	Thrombospondin 2
G06	Thbs3	1.148	Thrombospondin 3
G07	Timp1	0.66	Tissue inhibitor of metalloproteinase 1
G08	Timp2	1.0101	Tissue inhibitor of metalloproteinase 2
G09	Timp3	1.015	Tissue inhibitor of metalloproteinase 3
G10	Tnc	1.3592	Tenascin C
G11	Vcam1	0.4683	Vascular cell adhesion molecule 1
G12	Vtn	0.3833	Vitronectin
H01	Gusb	0.8326	Glucuronidase, beta
H02	Hprt1	0.9727	Hypoxanthine guanine phosphoribosyl transferase 1
H03	Hsp90ab1	1.0858	Heat shock protein 90 alpha (cytosolic), class B member 1
H04	Gapdh	1.0816	Glyceraldehyde-3-phosphate dehydrogenase
H05	Actb	1.0514	Actin, beta
H06	MGDC	2.9031	Mouse Genomic DNA Contamination
H07	RTC	1.2788	Reverse Transcription Control
H08	RTC	1.3031	Reverse Transcription Control
H09	RTC	1.3357	Reverse Transcription Control
H10	PPC	1.7061	Positive PCR Control
H11	PPC	1.7086	Positive PCR Control
H12	PPC	2.1558	Positive PCR Control
